# Parental intimate partner violence and abuse during the COVID-19 pandemic: Learning from remote and hybrid working to influence future support

**DOI:** 10.1177/17455057221129399

**Published:** 2022-10-12

**Authors:** Hayley Alderson, Simon Barrett, Michelle Addison, Samantha Burns, Victoria Cooling, Simon Hackett, Eileen Kaner, William McGovern, Deborah Smart, Ruth McGovern

**Affiliations:** 1 Population Health Sciences Institute, Newcastle University, Newcastle upon Tyne, UK; 2 Department of Sociology, Durham University, Durham, UK; 3 County Durham and Darlington NHS Foundation Trust, Darlington, UK; 4 Department of Social Work, Education and Community Wellbeing, Northumbria University, Newcastle upon Tyne, UK

**Keywords:** COVID-19, intimate partner violence and abuse, parents, lived experience, qualitative

## Abstract

**Objectives:**

The COVID-19 pandemic has exacerbated intimate partner violence and abuse. Incidents of intimate partner violence and abuse have increased as a result of household tensions due to enforced coexistence (multiple national lockdowns and working from home practices), economic stress related to loss of income, the disruption of social and protective networks and the decreased access to support services. This study aimed to understand how female survivors of parental intimate partner violence and abuse have experienced the adapted multi-agency response to intimate partner violence and abuse during the pandemic and consider learning from remote and hybrid working to influence future support.

**Method:**

This study adopted a qualitative research design, utilizing semi-structured interviews and a focus group. Data collection took place between March and September 2021. In total, 17 female survivors of intimate partner violence and abuse took part in the project; we conducted the semi-structured interviews via telephone (n = 9) and conducted an online focus group (n = 8).

**Results:**

Findings identified that services for those experiencing intimate partner violence and abuse need to be innovative, flexible and adaptable and ‘reach out’ to survivors rather than waiting for survivors to ‘reach in’ and ask for support. Findings show that the digital space highlights ‘missed opportunities’ for engagement with both professionals and peers and the potential for digital poverty is a key implication, which risks entrenching existing inequalities.

**Conclusion:**

In-depth consideration needs to be given to the design, delivery and evaluation of online interventions and provision of support to improve access and acceptability of services, maximize their effectiveness and to support the safety of survivors.

## Introduction

In the year ending March 2020, approximately 2.3 million (5.7%) adults aged 16–74 years in England and Wales experienced violence or abuse within the last year (1.6 million women and 757,000 men).^1^ Of these, 4.2% experienced abuse carried out by a partner or ex-partner, referred to as intimate partner violence and abuse (IPVA).^2^ The World Health Organization defines IPVA as ‘acts of physical aggression, psychological abuse, forced intercourse and other forms of sexual coercion, and various controlling behaviours such as isolating a person from family and friends or restricting access to information and assistance’.^3^ In addition, violence and abuse can take the form of debt bondage, intimidation, coercion, control, modern day slavery, forced isolation, physical, mental and sexual harms^4,5^ and is often closely connected to exploitation of those who are framed as vulnerable and/or ‘at risk’.^3^ IPVA is a prevalent and substantial concern that spans public health,^2^ child protection,^6,7^ criminal justice,^8^ health and social care and voluntary/statutory organizations. The Domestic Abuse Act which received royal assent on 29 April 2021 aims to,

raise awareness and understanding about the devastating impact of domestic abuse on victims and their families and to further improve the effectiveness of the justice system in providing protection for victims and survivors of domestic abuse and bringing perpetrators to justice.^9^

While IPVA is connected to multiple and persistent episodes of behaviour,^10^ there has been a surge in incidents reported through local police intelligence, voluntary and statutory agencies and calls to UK helplines^11^ during the COVID-19 pandemic. The incidence and severity of reported levels of IPVA increased around the world in response to various restrictions being imposed^12,13^ and work by Risser et al.^14^ showed overall increases in IPVA during the pandemic.

In the United Kingdom, measures such as mandating people to ‘stay home’, social distancing and isolation periods were introduced in March 2020, during the early stages of the COVID-19 pandemic to limit the spread of the disease. During the lockdown, restrictions led to the closure of centres and IPVA services, and most of the support transitioned to remote platforms and phone contact. While these enforced measures contributed to infection control and reduced the spread of the virus, they also played a role in the significant increase in psychological, physical and financial consequences for survivors and children experiencing violence within the home and exacerbated barriers to leaving an abusive relationship.^15^

While it is acknowledged that IPVA may have been occurring prior to the pandemic, it is recognized that incidents may be intensified as a result of household tensions due to enforced coexistence (multiple national lockdowns and working from home practices), economic stress related to loss of income, the disruption of social and protective networks and the decreased access to support services.^12^ This impact is felt most greatly as survivors may feel less safe to seek help while isolating within the home and it has been argued, via a gendered analysis, that a loss of a sense of control over lives and a sense of powerlessness may have led some men to seek to (re)assert masculine dominance at home.^16–18^ The intensified emotions experienced by survivors residing in close proximity to their abusers have resulted in heightened states of stress and anxiety being suffered, making the pandemic a much more dangerous time for women and their children.^19^

For parents, the additional factor of school closures put further strain on families, who were required to carry out home schooling and manage childcare responsibilities without any external support alongside their usual obligations.^20^ However, despite Piquero et al.’s^21^ systematic review and McNeil et al.’s^22^ rapid review reporting that school closures may have further increased tensions within families, at a time when children were exposed to parental IPVA or familial abuse at higher and more significant rates than previously, with greater frequency and intensity, these reviews report on prevalence and not narrative experiences. In addition, the amount of practical and emotional support that children access at schools via their peers and teachers as non-parental significant adults diminished,^11,23^ and the ability of professionals to detect levels of exposure to violence was limited.^24^ Childcare provided by the family’s wider support network (grandparents, friends, childcare providers) also reduced due to the restrictions, further enhancing the stresses of enforced coexistence. The combination of these factors impacted the safety of children experiencing violence within the family during the pandemic.^14^ Children who have been exposed to parental IPVA are significantly more likely than non-exposed peers to experience mental health problems,^25,26^ have lower educational attainment,^27^ experience IPVA in their own relationships and experience ill health^28^ all of which are aligned to constrained life chances.^28–30^ Many of these harms are often hidden however, and the true scale of parental IPVA is unknown. This is especially true within the current pandemic when incidents of violence and abuse may go unreported, as calling the police to intervene during lockdown may jeopardize the survivors safety further.^11^

Coming out of various phases of lockdown did not necessarily bring about a reduction in IPVA; for example, a recent Social Care Institute for Excellence report emphasized that, as social restrictions are lifted, perpetrators of IPVA may try to re-exert the control they perceive they had during lockdown by engaging in new and/or more harmful behaviour and intensifying coercive control.^31^ Substantial harms to the survivors, children and families associated with parental IPVA include social and psychological problems,^32^ physical ill health, poor mental wellbeing and financial problems for survivors.^33^

It is important to acknowledge that parents who are survivors of IPVA are not a homogeneous group; the intersections of identity are important to understand here^34^ as there is limited research that gives insight into IPVA^35^ and the varying impacts it has on marginalized parent groups,^36^ or how these parents are able to engage and access support, and whether support acknowledges intersections of identity, power and oppression.^37^ As such, this study adopts an intersectional lens via a ‘practical intervention in a world characterized by extreme inequalities’ (p. 785) to look at the way that gender interacts with other axes of identity such as race and class, how this affects the way that parents who are survivors of IPVA reflect on their experiences, and differing levels of engagement with support services.^35^

Despite there being multiple papers available regarding IPVA during the pandemic, there is still a scarcity of literature where parents who are survivors of IPVA are the primary focus of the research. Available literature often introduces parental survivors as a subcategory within the data and reports on prevalence rather than providing in-depth qualitative accounts of the experience of living through a pandemic while being exposed to IPVA and managing childcare responsibilities. This article aims to contribute knowledge regarding experiential accounts and focuses specifically on the lived experiences of parental intimate partner violence and abuse during the COVID-19 global pandemic, examining how the pandemic impacted upon survivors who are parents and how they experienced remote support. Furthermore, it also considers learning that can be taken from the delivery of remote support, and important considerations for practice when engaging with these parents, as services emerge from the COVID-19 pandemic and resume hybrid working.

## Methods

### Overview

This study adopted a qualitative research design; interviews were conducted between March and September 2021 and the focus group took place September 2021. A combination of purposive and a snowballing sampling framework was adopted, to recruit hidden populations into the study. An intersectional lens was adopted to analyse the data collected rather than shape the research design.^38^

### Participants

Participants were eligible for inclusion if they met the following criteria: a survivor of parental IPVA whom has accessed services during COVID-19, 18 years+, residing in the North East of England and able to provide informed consent. Exclusion criteria were as follows: a survivor who had not accessed services during COVID-19, below 18 years, residing outside of the North East of England and individuals who are unable to provide informed consent.

### Interview guide development

The topic guide design reflected the team’s involvement in previous research within the subject area and from conducting other sensitive research studies during the pandemic.

### Recruitment

In light of sensitive nature of the interviews, participants were recruited via gatekeepers. Gatekeepers consisted of individual professionals working on the frontline with survivors of IPVA (women’s refuge’s, voluntary/third sector services, local authorities). The gatekeepers introduced the research to potential participants and completed a consent to contact form that was shared with the research team if the participant agreed to be interviewed. This was a very important strategy to help maintain the safety of interested participants. If permission was acquired, a researcher then contacted potential participants, introduced themselves and talked through the participant information leaflet. All participants completed a consent form and emailed it to the researcher prior to commencing the interview.

### Data collection

It was envisaged at the beginning of the study that individual interviews would be conducted, as they would enable the research team to obtain a deeper understanding of an individual’s experiences regarding a sensitive topic. However, participants recruited through one organization requested that they could participate in a group as that felt more comfortable. Therefore, to respect the wishes of participants and be responsive to their needs, semi-structured interviews were conducted via telephone and a focus group via an online platform with survivors of IPVA. Semi-structured topic guides were chosen to enable the researcher to be flexible in their approach to exploring participants’ experiences and perspectives, while also having the scope to explore unforeseen areas of discussion.^39,40^ Interviews were organized at a time and date convenient to each participant. Participant safety was a key consideration when arranging interviews, whereby any concerns highlighted by gatekeepers were discussed and mitigated where possible. In addition, the safety of participants was checked at the beginning of the interview (e.g. they were asked who else was present within the home/environment they were in at the time of the interview), and it was agreed that if a participant needed to terminate a call for any reason, an agreed statement such as ‘I think you have the wrong number’ would be used and the researcher would attempt to re-connect with the participant later that day. If an interview was disconnected and contact could not be sought again later, the researcher would defer back to the gatekeeper and follow their established safeguarding protocols.

All interviews and the focus group were conducted in English; however, a translator was available within the focus group to assist with language needs when necessary. All interviews were audio-recorded. The focus group was not recorded at the request of the participants; however, notes of their discussion were taken, along with observations regarding how the women interacted with each other and discussed their experiences. Brief notes were made in relation to topics of convergence and divergences in information provided. In addition, detailed reflections were recorded immediately following the completion of the focus group by both researchers who facilitated the group.

The interviews and focus group were conducted, within North East England; participants were recruited via local authorities, women’s refuges and voluntary/third sector organizations. It was envisaged that approximately 20 interviews would be needed to achieve data saturation.^41^

Participants were given a gift voucher as recognition for bringing their expertise, knowledge and perspective to the research and subject area. Transcripts were anonymized, and all identifiable information relating to the participant sample was securely stored in a separate location. The study was approved by North West – Greater Manchester West Research Ethics Committee, 20/NW/0469.

## Qualitative analysis

All interviews were transcribed verbatim and subject to iterative, in-depth thematic analysis using an intersectional theoretical lens to make sense of the data. When analysing the interviews, we took an inductive approach, constantly comparing the interview transcripts to identify emerging themes.^42^ The reflective notes from the focus group were also compared to the transcripts. Two researchers (H.A. and S.B.) conducted the qualitative analysis. Verbatim quotes were used to highlight similarities and differences within the data and across participants. Trustworthiness of analysis and findings was ensured by discussing data among the wider team, inclusive of academics, practice partners and a survivor with lived experience to agree a consensus on the interpretations presented. The quotes included in this article came from survivors of IPVA, pseudonyms and anonymized participant numbers have been used throughout to protect each individual’s identity.

## Sample

In total, 17 female participants took part in the project; we conducted the semi-structured interviews via telephone (n = 9; eight White British and one Peruvian migrant) and an online focus group (n = 8; one British Indian, one British Pakistani, six Pakistani/Indian/Bangladeshi/Peruvian migrants with indefinite leave to remain/no recourse to public funds) with survivors of IPVA. Participants had between one and five children. All women self-identified as survivors of IPVA and at the time of interview were residing in refuge accommodation or away from the perpetrator, and for many, the move occurred during the pandemic.

The interviews were between 16 and 53 min in duration, with a mean time of 32 min and the focus group lasted 90 min. The analysis and extracts of participants’ reflections are explored in depth in the following.

## Results

### Impact of lockdowns

Survivors who had resided with their abusive partner during any part of lockdown described that they experienced increased forms of isolation, control and surveillance, which, in turn, impacted on their ability to access any support:

[Y]ou’re isolated. Well, I think they survive on that, because that’s what perpetrators do, they try and have you come away from your loved ones. So, it was kind of like a win-win situation. He always knew where I was, he always knew who I was talking to . . . So, it was like you’re even more isolated and you’re even more closed-off from means of support. (Participant 5, two children)

In practical terms, participants described that lockdown resulted in them experiencing increased anxiety and nervousness due to their abuser’s behaviour and being unable to seek their usual sources of support from family members due to isolation restrictions. This experience was common among survivors and was emphasized further for participant 1, who was not able to fly to see her family for a prolonged period of time:

I was very anxious and nervous as my family . . . couldn’t be here and we couldn’t get flights out to them so they said I would have to call the police because of the nature of what [my partner] was saying about me. (Participant 1, four children)

Participants also described how perpetrators used the social distancing restrictions to control them and enforce that they stayed at home, even when they were not adhering to the rules themselves:

It was Covid, but he does not want me to go out. He went to his friend’s house, but when I said, ‘I would like to meet these people that I [met on the internet]’, he said, ‘No. No, no’. Always, ‘No’, whatever I want is, ‘No, meeting is very dangerous’. (Participant 12, one child)

Being forced to spend more time with their partners was described by survivors as contributing to tension within the home, and participants stated that this was often associated with increased consumption of alcohol on behalf of perpetrators. This, in turn, was seen as a contributing factor to arguments and violence, and in some cases, the breakdown of relationships.

### Impact on the children

Impact on children was spoken about in two distinct ways, one being the direct exposure to instances of violent incidents. This was described as being intensified due to isolation measures resulting in parents being unable to hide IPVA and protect their children from witnessing it, as they were in the house more frequently and exposed to the abuse. Participants, whose children had been present and who had witnessed episodes of violence during lockdown, often described this experience as the catalyst for fleeing the family home and despite lockdown exacerbating barriers to leave abusive relationships, participants still made a choice to leave the relationship for their physical and mental health:

He was aggressive with me and he was always aggressive with me, and this time my daughter heard everything. All the fight. So, she asked me, ‘Mum, please leave’. (Participant 12, one child)

I thought I needed to stay with him for the children’s sake, but I couldn’t stay with him over the Covid, not good for the children’s mental health and probably all the other things. (Participant 8, five children)

Of equal concern to many survivors was the potential for re-traumatization of their children if they were discussing issues around IPVA via the telephone to professionals while their children were present. This was increasingly likely due to school closures throughout lockdown resulting in home schooling:

Because the Domestic Abuse Unit rang us, I couldn’t really openly tell them, because I had my seven-year-old [who was home schooling], who knows basically what I’m saying. So, I had to kind of like make it sound a bit better than I was feeling, so that she didn’t get concerned, if that makes sense? (Participant 2, one child)

This attempt to protect the children from overhearing details may have resulted in downplaying the full extent and impact of the abuse. This minimization and toning down of incidents potentially impacted on how an individual’s experiences and associated needs were understood and categorized in terms of severity, which, in turn, could impact on the levels of support offered.

One resource that was described as beneficial for survivors that were residing in refuges at the time of the interview was the availability of a creche service. The opportunity for survivors to have their children looked after in a safe environment and have protected time to obtain support, without their children present was appreciated:

You can do all your meetings and appointments and stuff, if need be, in that time. So, I used to get my support plan– like my support meeting would always be scheduled in when the little one was in the crèche, just so you’re not having to talk about all of this stuff, in front of the kids. (Participant 21, two children)

### Survivors contact with police

Several participants reported having contact with the police during lockdown. Contact with the police regarding in-person visits, advice and signposting to other support services and providing updates was generally reported by survivors in a positive manner:

They sent out a woman police officer the next day and she was lovely . . . when I told her what was happening, she said you are doing all the right things . . . she put me at ease . . . She gave me the confidence to lift the phone to them if he started again and he did. (Participant 1, four children)

Most of the contact with the police occurred through phone calls. However, despite this more remote method of communication survivors reported that they felt the police had a heightened awareness of the potential impact of lockdown on incidents of IPVA and they responded sensitively. The survivors described feeling a sense of validation that their concerns were being taken seriously and felt satisfied with available safety measures that were implemented during the pandemic:

I think they knew like, if he came to my house this time I couldn’t really leave, because we’re in lockdown . . . this time they actually searched my house and my garden, and they were doing walks around my street to make sure if he came, before they arrested him, that I was safe in my house . . . I think the way they handled it, I think it was more down to Covid, because I was locked in the house. The responding officers who came out first, they were a lot, like they cared more, and they were constantly reassuring us and ringing to make sure I was okay. (Participant 2, one child)

### Women’s refuges

Participants described varied experiences of women’s refuges during lockdown. For some, the refuge was a place that provided everything that they needed, both physically in terms of shelter and housing and also emotional support too. They provided much needed support during COVID-19 that many survivors could not receive elsewhere:

They [refuge staff] have sorted my housing application form out, they’ve referred me to the Adult Services, they are trying to help me to get food parcels and things like that. Because my last wage, in June, I didn’t get a payment off the dole last month, so I’ve gone eight weeks with no food and stuff like that. So, they have given me a lot of support in the women’s refuge, they have done a lot for me. (Participant 11, four children)

Others described experiences which they felt were traumatizing and sometimes worse than the situation they had sought to escape. Survivors described conflicts with other residents within the refuge, while this may be true prior to the pandemic, the dynamics between survivors within the refuge during COVID-19 was intensified due to women feeling isolated within their own accommodation and/or tensions between women who were not seen to be following social distancing restrictions:

I felt totally unsafe in the refuge to the fact that they had to move us. There was nothing put into place . . . none of the policies were robust enough at all. (Participant 9, one child)

Survivors were restricted in their ability to leave the refuge and obtain support from family members, as would have happened if travel restrictions were not in place:

I hate it [at the refuge], I do, I’ll be honest, I don’t like it. I feel I’ve got more hassle here than I did in the relationship. The bitchiness . . . It’s just ridiculous, honestly. I was crying on the phone to my mum . . . begging her for me to come back there . . . we’re all in here for the same reason, we should all be helping each other, not taking your anger out on somebody else. (Participant 11, four children)

Participants explained that they had experienced a delay in receiving a full package of support, such as access to therapeutic support due to the pandemic restrictions and associated additional childcare responsibilities:

Respondent: I just want to get my life back on track.

Interviewer: What sorts of things have they been doing to try and help you do that?

Respondent: At the moment, not a great deal, but I think we’ll just wait until the kids are in school, so they’ll get more time with me. (Participant 8, five children)

### Access to IPVA support

Survivors reported receiving specialized support from various services and agencies during lockdown, including women’s shelters, social workers, the justice system, survivor support services and local schools. Participants expressed appreciation for the positive impact of this new network of support received during the COVID-19 pandemic:

They (Police) called up the domestic violence team . . . You are assigned a [Domestic Violence] worker and they ring you up every couple of days or you can ring them whenever. She was brilliant. It was them who helped me through when actually he kept the kids. (Participant 1, four children)

There was recognition from participants that remote methods of engagement resulted in professionals having the flexibility to engage with survivors more frequently due to reduced amount of time being taken to travel between appointments:

I think maybe the online stuff can be good as well. So, if you’ve got somebody with a massive caseload who is really busy, at least it might give them an opportunity to check in with somebody every week [online] for 15 minutes when they couldn’t have the time that week to go and visit them. (Participant 9, one child)

For many, the support they received was viewed as vital, and this often took the form of one agency or often one individual, with whom they had a good relationship, being able to connect them to other services that could provide help and advice:

She [keyworker] was just really understanding. She was just lush. I’m gutted she has left, to be honest. She was so nice. (Participant 21, two children)

Interview participants highlighted the flexibility and adaptability of specialist IPVA programmes during COVID-19 as a key feature of support. When support services moved online, this was often reflected upon by the women in ambivalent terms. For some participants, this transition was a smooth one, with no obvious disruption or downsides:

Before, I used to be there [at the domestic abuse service] three days a week, doing different courses and that. Then obviously lockdown happened, but they still kept everything, as it was, but we just went on[line] and did it all. (Participant 2, one child)

However, other participants spoke of barriers and added complications that occurred because of the transition to online and telephone support. Unsurprisingly, the lack of face-to-face interaction with another human being was the most common downside to online services described by survivors:

There’s like an energy in the room that you don’t get online . . . If you’re in a room with people and you’ve got a therapist working, they can sense when something’s wrong with somebody, they can have a word with them after, and you don’t have that on[line]. It’s just, you’re finished on[line], you all log off and go about the rest of your day, don’t you? (Participant 9, one child)

The lack of human interaction did have serious consequences for some survivors, and rather than providing help, these online support groups were the cause of emotional distress:

One of the times I was online, I just cried the entire way through it, but nobody recognised that. I had – and that triggered all my nightmares, I had nightmares and that but nobody . . . whereas had I been in the class, that would have been spotted. (Participant 10, four children)

The remote or online platforms could be seen as inhibiting the rapport building that would occur if support was taking place face to face:

it’s not as easy to talk to someone over the phone as it is face to face, I think . . . Because obviously you don’t know who you’re talking to over the other end of the phone, you can’t see their face or anything, you can’t get to know them, to open up to them. (Participant 11, four children)

There were also practical and systemic issues which led to problems when trying to access therapy online. Participant 10 describes waiting to receive Eye Movement Desensitization and Reprocessing (EMDR) which is a form of therapy to support her with anxiety and post-traumatic stress disorder developed through experiencing IPVA:

I waited for a year and a half for complex post-traumatic stress [therapy], and then when it came along, with it being the pandemic, we tried to do it online and it wasn’t really working. And then my sessions had ran out. So, then I started the queue again . . . I kept saying to the therapist, like, she couldn’t understand, she didn’t know if it was like my broadband, her broadband . . . but that wasn’t really helpful to me because it just – I had been waiting for a year and a half for this therapy and then the therapy came, and I couldn’t meet anyone eye to eye anyway. (Participant 10, four children)

This emotional distress and frustration for participants centred around the lack of flexibility regarding session delivery, that is, despite not being able to fully engage in the EMDR therapy due to Internet connectivity issues, participant 10 had received her quota of sessions and was effectively closed to this treatment. Some survivors felt defeated and unable to access the help they needed during the pandemic, a situation often exacerbated by reduced levels of confidence resulting from coercive control which abusive partners had exerted over these survivors’ lives, and their previous experiences of trauma inducing violence and abuse:

I have a lifetime of being beaten up . . . I’ve tried to kill myself God knows how many times . . . I’m at the end of my tether, I get where I feel defeated and I think, ‘What’s the point?’ because I don’t know what to – I’m ringing people. There’s nothing open. I’m trying to figure it out on my own and I don’t know where to go . . . I’m full of self-doubt. I don’t believe in myself. I don’t have any confidence. (Participant 10, four children)

### Specialist support for ethnic minority survivors

Minoritized women in the study reported varying experiences of IPV during lockdown; they described facing additional pressures due to intersections of race, gender, class and their immigration status. All focus group participants had received support during lockdown from an organization for Black and minoritized women focusing on the intersection of race and gender. The centre provided intersectionally designed practical support around securing an income, immigration advice, night-time emergency support, housing advice and during lockdown a food bank was available. While they did not report that lockdown had any impact upon the services they received, it is important to recognize that this was the first time each of them had accessed such support.

Participants spoke of the lack of social or support networks outside of their own or their partner’s family, and how coercion and control were often exerted by the wider family unit. As well as aggression from partners and families, fears of stigma and shame and honour-based violence were used (or threatened) in an attempt to influence the women to remain with their abusive partners. This was intensified during COVID-19 when they experienced stricter controls on their freedom due to family members being more frequently present within the home due to lockdown restrictions. Most of these women (n = 7) spoke of the amplifying effect of intersectional harms related to the threat of deportation, insecure or uncertain visa situations, and language as a barrier to accessing support, as well as concerns that the conditions of their entry visa meant they were not allowed to access public funds while in the United Kingdom. This is exemplified as follows:

I did not know that in this country someone could help me. I did not know that. I was two months going around asking people . . . because I did not have anyone here. I did not know the rules in this country. I did not know that anyone can believe me. I did not know anything. (Participant 12, one child)

## Discussion

Findings from this study highlight that there is a need for survivors exposed to IPVA to re-engage with and maintain social connectedness, especially during times of enforced isolation. Many of our findings are pertinent to all survivors of IPVA; however, it needs to be acknowledged that COVID-19 had an uneven impact on how parents experiencing IPVA engaged with and accessed support as the pandemic prevented face to face access to both familial support and professional services. Reduced access to support networks was problematic as the previous literature has identified that regular contact with friends, family and professionals can support healing from abuse.^43^ As identified in the previous literature, the government-imposed restrictions closed down routes to safety for many survivors of IPVA and their children inducing greater harms, particularly at the intersection of race, gender and class, and those with a precarious immigration status. For some, this resulted in their children being exposed to more severe violence and at an increased frequency, due to extended periods of time when they were present within the home.^14,22,23,44^ As we attempt to re-establish ‘normality’ post the COVID-19 pandemic, it is important for services to consider an intersectional approach to support survivors to help sensitively reconstruct their support networks.

In line with the available literature, for survivors still residing with their partners, this study highlights how lockdown restrictions could enable perpetrators to exert further coercive control mechanisms, including increased levels of isolation, control and surveillance.^45^ This study has further highlighted the use of confinement and the threat of contracting the virus as an additional mechanism to facilitate their abuse by perpetrators.^15^ While the issue of digital monitoring was not discussed explicitly within the our sample, the literature shows that accessing support via online methods can be challenging due to perpetrators not allowing survivors access to their phones or conversely perpetrators using tactics such as digital monitoring and tracking as a form of coercive control^46–48^ both resulting in limited access to services. Available literature shows that the transition to virtual support increased concerns for frontline providers regarding the safety of survivors and that modes of communication were adjusted to address privacy concerns for survivors still residing with their abusive partners.

The response to the COVID-19 pandemic has led to new ways of working, and accelerated a move towards online and virtual support;^49^ some of which may continue post pandemic. Recent studies found that from a service provider/advocate perspective, the transition to virtual support provided both challenges and opportunities.^50^ Participants explained that organizations often reacted rapidly and adapted their service to offer continued support online and over the phone, which was greatly appreciated by many survivors. Police were described as having a heightened awareness of the potential intensification of domestic violence incidents due to prolonged periods of isolation and were sensitive to the needs of survivors,^51^ this was of particular importance to women who were considering the safety of their children as well as themselves. The requirement for police to respond differently was acknowledged, and within a review of policing during the pandemic, it is reported that police forces recognized that they needed to work innovatively and had to ‘reach in’ to survivors rather than waiting for them to ‘reach out’.^52^ Furthermore, it has been reported that during the pandemic, many police forces increased their use of Domestic Violence Protection Orders which can prevent the perpetrator from returning to a residence and from having contact with the survivors for up to 28 days.^52^ These increasingly pro-active methods of service provision will be beneficial as one mechanism to contribute to the prevention of violence, abuse and intimidation that disproportionately affects women and girls.

Participants explained that some services responded in an innovative and flexible way to continue to meet the identified needs of survivors and their families. For some participants, there were clear benefits of support being remote, such as the obvious reduction in travel time and associated expense to attend appointments, this was in keeping with available literature^49^ and was of particular importance to individuals with childcare responsibilities. An additional key driver of perceived success of online working was a good connection in terms of Internet provider and also a good connection in personal relationship with a kind, supportive, friendly professional to help individuals navigate the complex systems of support.

However, this article highlights that the move to online and/or remote methods of engagement came at a cost to some survivors who felt a loss of positive interaction with peers or practitioners. This was a view shared by frontline workers who identified it was difficult to build relationships and trust virtually.^50,53^ Online platforms could hamper the ability for professionals to pick up on body language and could result in overlooking emotional distress. A number of important factors influenced the effectiveness of online/remote provision inclusive of access to a safe and confidential space to engage with support,^48,54^ challenges establishing a therapeutic relationship and difficulties communicating emotions and empathy.^55^ When referring to online support, terms such as being ‘a box on a screen’ and ‘logging off’ at the end of the session were used, implying more dehumanized methods of engagement. In addition, online platforms reduced the opportunity to engage in genuine peer-to-peer interaction and support that may have been available if services had taken place face to face. This felt like a missed opportunity for some individuals who wished to develop a support network with other survivors and engage on a more therapeutic level with peers with lived experience.^56^ Despite these concerns, a number of studies have reported that a therapeutic alliance can be established online^57,58^ and that patients can experience online support positively when delivered well.^59^

There was also a practical issue of accessibility due to available Wi-Fi networks, when these facilities did not work as hoped it led to frustration and disruption, especially in form of therapy such as EMDR which as a form of psychotherapy relies on the therapist being able to clearly observe an individual’s eye movement. The potential for individuals (professionals and service users) to experience technical difficulties accessing support and/or interruptions to Internet connect within sessions need to be taken into consideration when delivering interventions and support.^54^ In addition, the issue of digital poverty and digital inequalities has the potential to widen health inequalities and alienate those who cannot access services in this way.^60^ Service providers overlooked the intersection of gender and class, amplifying harms for women who were also in poverty and those experiencing digital poverty became further marginalized due to transitioning services online which certain parents could not easily access.^53,60^ Services not only need to be mindful of privacy concerns when attempting to engage remotely with survivors but also how online services can exacerbate harms experienced at the intersection of class and gender as individuals become even further removed from accessing support.^61^

Minoritized survivors experienced additional complexities. The unstable immigration status and the threat of deportation alongside the intensified levels of coercion and control experienced within the extended family network during COVID-19 exacerbated already difficult circumstances.^62^ While these issues were present prior to the pandemic, COVID-19 has potentially exacerbated the ‘justice gap’ as it was recognized that refuge bed space for Black and minoritized women was limited during the pandemic.^63^

Survivors residing in women’s refuges also reported varying experiences, ranging from positive experiences within which women felt their holistic needs were being met, through to increasingly negative experiences due to relationship dynamics within the refuge environment.^64^ This divergent set of encounters highlights that services may benefit from adopting an intersectional approach to service provision to meet the needs of their service users. The additional pressure of refuge services having to be restructured to adhere to social distancing restrictions will undoubtedly have exacerbated an already stressful environment^65^ for survivors residing there with children and having limited capacity to utilize shared facilities.

While experience of support during COVID-19 varied, what was constant was the presence of structural, systemic and complex barriers to accessing support which need to be negotiated. This navigation of support requires persistence and determination, a situation which was often exacerbated due to the fact that most of those needing help may have low self-confidence and low self-esteem due to experience of coercive control and perpetrator imposed isolation.^66^ Mental health needs around anxiety, depression and post-traumatic stress disorder should be considered for survivors of IPVA.^67,68^ The COVID-19 pandemic has seen a huge rise in the prevalence of mental health challenges as survivors have been forced to spend increased amounts of time with their abuser.^16^ A high proportion of individuals experiencing IPVA report multiple abusive relationships including witnessing and being a survivor of abuse during childhood. In many cases, survivors explained that due to sharing parental responsibility, ending the relationship did not automatically result in abuse ceasing. Instead, perpetrators were described as relentlessly reminding and retraumatizing the victim repeatedly through shared parenting. This cyclical and ongoing nature of abuse requires services to take a trauma informed approach to survivors.^69^ Much work needs to take place post pandemic to start addressing the mental health needs of survivors that remained unmet during COVID-19.

## Strengths and limitations

The strengths of the study are that findings are current and salient as we emerge from the COVID-19 pandemic. The qualitative interviews provide rich accounts of parents affected by IPVA who experienced service provision during the pandemic and highlight areas of consideration for service providers as hybrid working structures are introduced.

The limitations are that the study was set in the North East of England and issues may not be the same as other areas in England. In addition, gatekeepers were used, which could potentially have introduced a bias to the participants recruited. However, participants reported varied experiences of service provision which was reassuring.

While the small, varied sample size is within usual range for in-depth qualitative studies and was sufficient to examine the main analytic themes of the impact of lockdowns, the impact on children, access to IPVA support and women’s refuges, the sample did not allow data saturation among subgroups such as immigrant versus non-immigrant participants.

## Implications for policy and practice

Several implications for policy and practice have been identified. The move to remote support has highlighted both negative (restricted ability to engage openly due to children/perpetrator being present, safety risks) and positive consequences (flexibility, less travel, more economical). Organizations providing specialist support (e.g. children’s services, voluntary and third sector, local authorities) should consider the feasibility of delivering intersectionally designed support and interventions using a mixture of face-to-face appointments to build rapport and remote measures (online video platforms, telephone calls) once a relationship has been established to provide flexibility.

Participants within this project identified challenges of accessing online groupwork courses. Therefore, we propose that groupwork delivered to survivors should be delivered face to face wherever possible to optimize the impact of the content being delivered and facilitate an environment where peer support can be utilized.

A further implication highlighted within this project relates to amplified harm at the intersections of race, gender, class and immigration status, particularly exemplified in the experiences of minoritized women with indefinite leave to remain/no recourse to public funds. It would be beneficial to take an intersectional lens and consider how a survivors’ identity as a non-English speaking, immigrant could lead to a continuation of oppressive experiences when attempting to access support for IPVA. We suggest that further awareness regarding the Destitution Domestic Violence concession is needed among service providers and the police; specialist culturally sensitive support needs to be more easily accessible and designed with intersections of power and oppression in mind and accessing independent translators rather than family members are required to maximize the potential for marginalized survivors to receive the necessary support.

## Conclusion

This study has provided valuable insights into the experiences of participants accessing support during COVID-19. Support services for parents experiencing IPVA need to be innovative, flexible and adaptable and ‘reach out’ to survivors rather than waiting for survivors to ‘reach in’ and ask for support. In-depth consideration needs to be given to the design, delivery and evaluation of online interventions and provision of support to improve access and acceptability of services, maximize their effectiveness, reduce harm, and to support the safety of survivors. Findings show that the digital space highlights ‘missed opportunities’ for engagement with both professionals and peers and the potential for digital poverty is a key implication, which also risks entrenching existing inequalities that are amplified by intersections of race, class and gender. Further work to establish who is ‘invisible’ to services because they do not have access to a phone or to data is necessary.

## Supplemental Material

sj-docx-1-whe-10.1177_17455057221129399 – Supplemental material for Parental intimate partner violence and abuse during the COVID-19 pandemic: Learning from remote and hybrid working to influence future support Click here for additional data file. Supplemental material, sj-docx-1-whe-10.1177_17455057221129399 for Parental intimate partner violence and abuse during the COVID-19 pandemic: Learning from remote and hybrid working to influence future support by Hayley Alderson, Simon Barrett, Michelle Addison, Samantha Burns, Victoria Cooling, Simon Hackett, Eileen Kaner, William McGovern, Deborah Smart and Ruth McGovern in Women’s Health

sj-docx-2-whe-10.1177_17455057221129399 – Supplemental material for Parental intimate partner violence and abuse during the COVID-19 pandemic: Learning from remote and hybrid working to influence future support Click here for additional data file. Supplemental material, sj-docx-2-whe-10.1177_17455057221129399 for Parental intimate partner violence and abuse during the COVID-19 pandemic: Learning from remote and hybrid working to influence future support by Hayley Alderson, Simon Barrett, Michelle Addison, Samantha Burns, Victoria Cooling, Simon Hackett, Eileen Kaner, William McGovern, Deborah Smart and Ruth McGovern in Women’s Health

## Declarations

**Ethics approval and consent to participate:** The study was approved by North West – Greater Manchester West Research Ethics Committee, 20/NW/0469. All participants provided written consent via email prior to the interview.

**Consent for publication:** Informed consent for publication was provided by participants. Non-essential identifying details have been omitted.

**Availability of data and materials:** Data could be requested from the corresponding author on reasonable request.
