# Supplementary material for: Parental intimate partner violence and abuse during the COVID-19 pandemic: Learning from remote and hybrid working to influence future support
Source: Womens Health (Lond). 2022 Oct 12;18:17455057221129399. doi: 10.1177/17455057221129399 (PMC9557270; doi:10.1177/17455057221129399)
Supplement: sj-docx-1-whe-10.1177_17455057221129399 – Supplemental material for Parental intimate partner violence and abuse during the COVID-19 pandemic: Learning from remote and hybrid working to influence future support [file sj-docx-1-whe-10.1177_17455057221129399.docx]

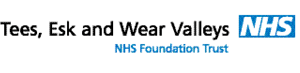

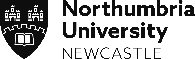

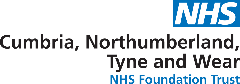

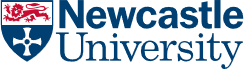


PROTECT COVID 19

Topic guide

***Thank you for taking part in this study of Parental IPVA. I would like to ask you about your experiences of the support services you received regarding parental IPVA during the COVID 19 Pandemic. I will use your ideas to feed back to multi-agency partners to highlight what they are doing well and what may need to be improved.***

***Reiterate issues of confidentiality and anonymity, the purpose of the study and what is going to happen to the data.***

***Complete Consent Forms***

***Switch on audio recorder (if participant has consented)***

***Interviewer- We need to think of lockdown as March- May 2020 and January 2021 to current date and socially distancing restrictions as May- January 2021. . It would be good to capture data on both time points if possible***

Please could you tell me a little about your current family situation (Probe: living arrangements, number of children/childcare responsibilities)

Can you tell me about who you have got in your life currently that supports you? (Probe: family, friends, professionals)

**I’m going to ask you some questions about Intimate Partner Violence, I just wanted to check that this is okay, and you are ready?**

Can you tell me about your perception of parental intimate partner violence? (Probe: Are you currently experiencing parental IPVA? How long has this been occurring?)

Has COVID impacted on your experiences of IPVA? In what ways? (Probe: frequency, type of IPVA, children being present?)

Can you tell me if you had contact with services regarding parental IPVA prior to the COVID lockdown? (if so which agencies and what was your experience of the support/contact you received)

Please could you also describe to me your experience of the responses you have received regarding IPVA during COVID 19? (probe: are they the same/different to pre lockdown services/responses)

How did practitioners improve your situation around Parental IPVA? Can you describe some of the things that they did? (Probe: what support was offered to you and your children?)

Did anything involving services/practitioners make your situation worse around parental IPVA? Can you describe some of these instances and what happened?

Can you tell me the impact that the approach practitioners used, and the strategies used during COVID 19 had upon yourself and any children exposed to parental IPVA? (probe: think about risk/safety/mental health and wellbeing)

Can You tell me how involvement with services made you feel? (how did this affect their engagement)

Can you tell me what you think were the barriers to engaging with multi-agency partners regarding IPVA during the pandemic? (probe: partner/children being present)

Can you also tell me what worked well during the pandemic that could be taken forward into standard practice? (probe: remote methods of working)

Is there anything else you would like to add?

***Turn audio recorded off***

***Thank participants for their time, explain what will happen next with the data and reiterate confidentiality.***
