# Supplementary material for: Parental intimate partner violence and abuse during the COVID-19 pandemic: Learning from remote and hybrid working to influence future support
Source: Womens Health (Lond). 2022 Oct 12;18:17455057221129399. doi: 10.1177/17455057221129399 (PMC9557270; doi:10.1177/17455057221129399)
Supplement: sj-docx-2-whe-10.1177_17455057221129399 – Supplemental material for Parental intimate partner violence and abuse during the COVID-19 pandemic: Learning from remote and hybrid working to influence future support [file sj-docx-2-whe-10.1177_17455057221129399.docx]

COREQ guideline for IPVA.

| **No** | **Item** | **Guide questions/description** |
| --- | --- | --- |
| **Domain 1: Research team and reflexivity** |  |  |
| Personal Characteristics |  |  |
| 1. | Interviewer/facilitator | HA, SB, SBu and HF (not included in the paper due to retirement) conducted the interviews. |
| 2. | Credentials | HF is a social worker based within a local authority. HA, SB (male) and SB (female) all have a PhD and have extensive experience of conducting qualitative interviews with vulnerable and marginalised populations. |
| 3. | Occupation | HA is a Senior Research Associate and an ARC Embedded researcher within the Children and Families theme.  SB is a Research Associate.  MA is an Assistant Professor.  SB is a lecturer at Durham University.  VC is a Specialty Registrar in Public Health.  SH is a Senior Clinical Lecturer in Mental Health  EK is Professor of Public Health and Primary Care Research.  WM is a Senior Lecturer.  DS is a research assistant.  RM is a Senior Lecturer in Public Health Research. |
| 4. | Gender | The researchers conducting the interviews were female and male. |
| 5. | Experience and training | All researchers had extensive experience of conducting qualitative research and working on multiple studies with vulnerable participants. |
| Relationship with participants |  |  |
| 6. | Relationship established | Relationships were not established prior to study commencement. |
| 7. | Participant knowledge of the interviewer | Participants knew where the researcher was based and the purpose of the study. |
| 8. | Interviewer characteristics | The gatekeepers and interviewers reported the reasons why the research was important. |
| **Domain 2: study design** |  |  |
| Theoretical framework |  |  |
| 9. | Methodological orientation and Theory |  |
| Participant selection |  |  |
| 10. | Sampling | Participants were recruited using purposive and snowball sampling. |
| 11. | Method of approach | Service users were approached using a mixture of face to face and telephone contact by gatekeepers of frontline services inclusive of local authorities, refuges, and domestic violence support services. Due to Covid contact between the researchers and the service users always took place via telephone. |
| 12. | Sample size | 17 survivors took part in the qualitative interviews. |
| 13. | Non-participation | Participants volunteered to take part in an interview. Non-participation occurred if individuals declined to take part or elected not to attend the interview. |
| Setting |  |  |
| 14. | Setting of data collection | Due to interviews taking place via the telephone. Interviews with survivors took place when the participants were in a safe location of their choice. Researchers ensured that they were in a room that enabled the interview to remain confidential and uninterrupted. |
| 15. | Presence of non-participants | All survivor interviews reported in this paper took place with only the participants and researchers present. |
| 16. | Description of sample | All participants resided within the North East of England.  They were 18 years + and were currently accessing/had recently accessed support regarding IPVA. |
| Data collection |  |  |
| 17. | Interview guide | Semi structured interview guides were used by the researchers. |
| 18. | Repeat interviews | Repeat interviews were not conducted. |
| 19. | Audio/visual recording | All the interviews were audio recorded.  The focus group was not recorded at the request of participants |
| 20. | Field notes | Field notes were made following the interviews and focus groups. |
| 21. | Duration | The duration of interviews varied from 16- 53 minutes.  The focus group lasted 90 minutes. |
| 22. | Data saturation | Data collection continued until data saturation was reached regarding the salient themes such as the impact of lockdowns, the impact on children, access to IPVA support and women’s refuges. However, the small sample size did not allow data saturation among subgroups such as immigrant v non-immigrant participants. |
| 23. | Transcripts returned | No. |
| **Domain 3: analysis and findings** |  |  |
| Data analysis |  |  |
| 24. | Number of data coders | 2 Researchers coded the data. |
| 25. | Description of the coding tree | The coding tree was discussed within qualitative data meetings within the study team. |
| 26. | Derivation of themes | Thematic analysis was conducted. |
| 27. | Software | NVIVO software, was used to help manage the Qualitative data. |
| 28. | Participant checking | This did not occur. |
| Reporting |  |  |
| 29. | Quotations presented | Yes participant quotations were used to illustrate themes/findings. Quotes were identified using pseudonyms and a descriptor i.e., *Participant 5, 2 children* |
| 30. | Data and findings consistent | Yes there are consistency between the data presented and the findings |
| 31. | Clarity of major themes | Yes major themes are clearly presented in the findings |
| 32. | Clarity of minor themes | No as this was not relevant within this paper. |
